# Supplementary figures and images for: Characterization of intestinal O-glycome in reactive oxygen species deficiency
Source: PLoS One. 2024 Mar 14;19(3):e0297292. doi: 10.1371/journal.pone.0297292 (PMC10939276; doi:10.1371/journal.pone.0297292)

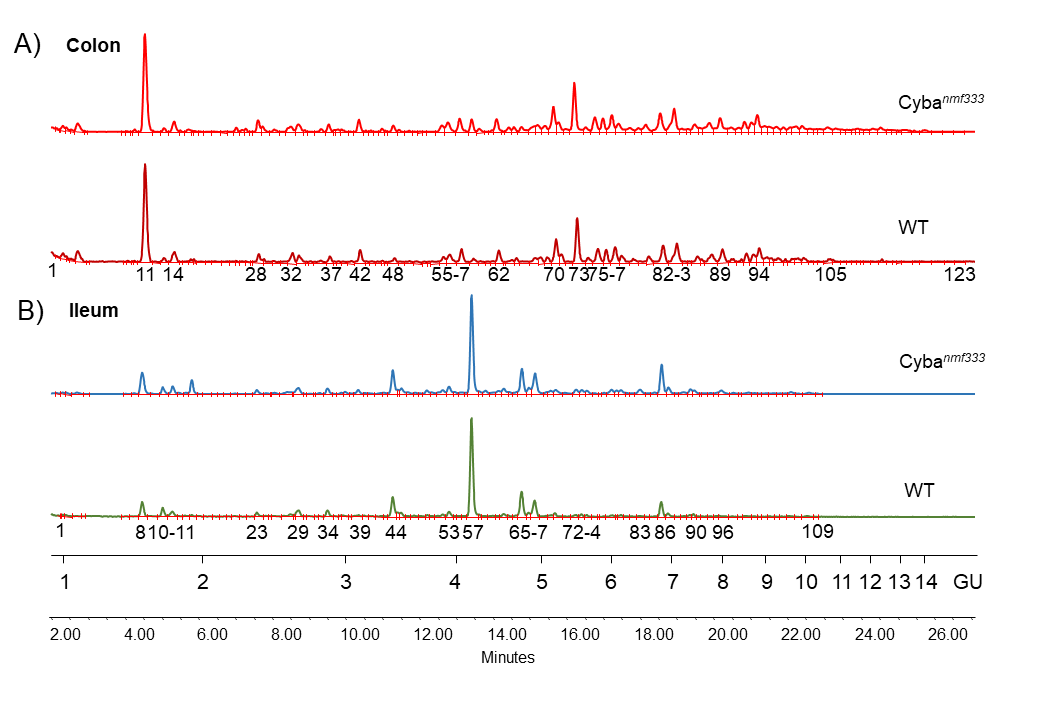

Supplement: S1 Fig — (TIF) [file pone.0297292.s001.tif]
