# Supplementary material for: Characterization of intestinal O-glycome in reactive oxygen species deficiency
Source: PLoS One. 2024 Mar 14;19(3):e0297292. doi: 10.1371/journal.pone.0297292 (PMC10939276; doi:10.1371/journal.pone.0297292)
Supplement: Attachment — Submitted filename: Response letter.docx [file pone.0297292.s006.docx]

Dear Editors,

We thank the reviewers for their helpful and constructive suggestions on our paper entitled:- “***Characterization of intestinal O-glycome in reactive oxygen species deficiency***”.

We hope that both you and the reviewers find this revised manuscript improved and now acceptable for consideration for publication.

The revised draft of the manuscript along with the comments in this letter addresses the concerns raised by the reviewers. Our specific point-by-point responses are found below and we have numbered the reviewers' queries to assist in the review process.

Additional requirements were addressed, namely-

Style was updated according to the instructions, typos were corrected.

Remaining data were deposited to Glycopost project ID no. GPST000386 (https://glycopost.glycosmos.org/entry/GPST000386).

The funders had no role in study design, data collection and analysis, decision to publish, or preparation of the manuscript.

**Response to Editor and Reviewers**

Response to Editor

1)*Why did you sacrifice after 14-16 weeks?*

We sacrificed older mice as recommended for mucus analysis as mouse mucus composition has stabilized after 12 weeks of age.

2)*Indicate arrows on the figures*

As requested, we have added arrows for goblet cells and the MUC2 layer and indicated this in the Figure legend Fig 1.

3)*Has the UEA been confirmed on how many slides? Have gender differences been highlighted?*

UEA staining was performed on colonic tissues of 4 mice/group (both genders, 3-4 slides with 4 tissues per slide/mouse) and representative images are shown. The *Cyba^nmf333^* mice were thoroughly characterized in Mucosal Immunol. 2019, 12(6):1316-1326 using female and male mice, and no apparent gender-specific differences were observed in mucus (Fig.4, Figs S6, S7). Therefore, as indicated, mucus was harvested from both genders and combined for analysis.

Response to Reviewer 1

1)*Line 202, correct "Ffollowing" and insert a period before.*

The text has been corrected throughout.

2)*Fig 1 would be clearer if some arrows were inserted to indicate goblet cells and MUC2 layers.*

As requested, we have added arrows for goblet cells and the MUC2 layer and indicated this in the Figure legend Fig 1.

3)*Fig. 3 A,C are too small and characters cannot be read, they should be larger*

The small characters on Figure 3A and C were increased in size.

Response to Reviewer 2

1)*Authors will need to review typos and punctation*

The text has been corrected throughout.

2)*Have you noticed any differences in mucin expression between genders?*

The *Cyba^nmf333^* mice were thoroughly characterized in Mucosal Immunol. 2019, 12(6):1316-1326 using female and male mice, and no apparent gender-specific differences were observed in mucus (Fig.4, Figs S6, S7). Therefore, as indicated, mucus was harvested from both genders and combined for analysis.

Kind Regards,

Radka Saldova et al.
